# Supplementary material for: Tribological Properties of Nitrate Graphite Foils
Source: Nanomaterials (Basel). 2024 Sep 15;14(18):1499. doi: 10.3390/nano14181499 (PMC11434555; doi:10.3390/nano14181499)
Supplement: Supplementary file 1 [file nanomaterials-14-01499-s001.zip › X-ray data/misorientation/01Hz/over500/dens_16/RD/222.pdf]

Raw data

Sample Name:			X-ray	:	Cu / 40 kV / 30 mA	Counter	:	Scintillation counter
File	:	222.raw	Goniometer	:	Ultima IV			
Comment	:		Attachment	:	MPA-ML4(with gamma) for stress			
Date	:	May-17-24 10:23:12	Filter	:	K-beta filter	Scan mode	:	Continuous
Operator	:	Administrator	I.Monochro	:	CBO	Scan speed	:	5.00 deg./min.
			C.Monochro	:	Not installed	Sampling width	:	0.05 no unit
			DivSlit	:	Open	Scan axis	:	theta
			DivH.L.Slit	:	10.00mm	Scan range	:	10.00 -> 80.00 no unit
			SctSlit	:	2/3?‹|2/3deg.	Fixed angle	:	0.00 no unit
			RecSlit	:	0.30mm			

Memo :

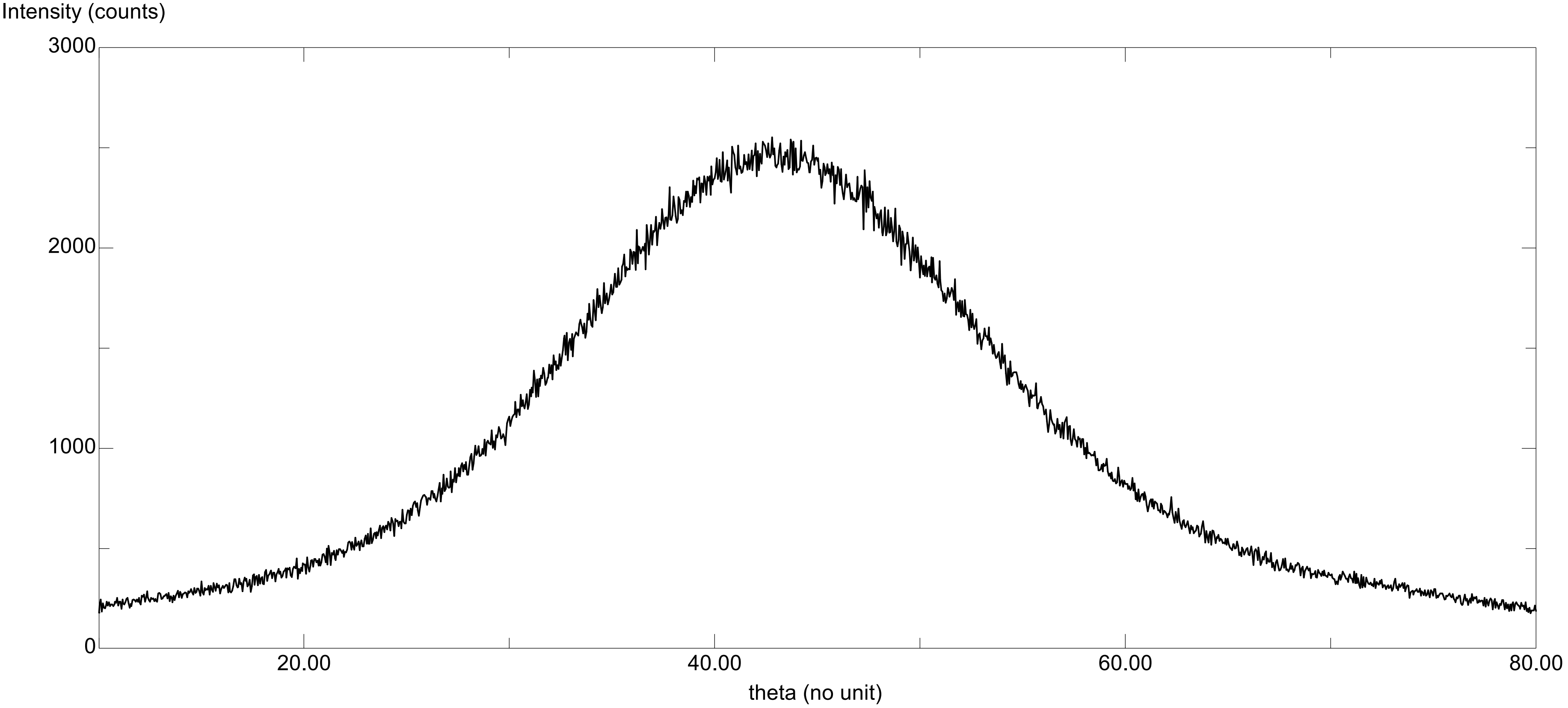

Integral Int. Calculation (History)

Sample Name: File : 222.raw Date : May-17-24 10:23:12 Operator : Administrator

Comment : Memo :

Peak calc. method: FW2/3M

h	k	l	Start	Stop	Peak	d-value	FWHM	Max. int.		Gross int.	Integ.w	Integ. int.
0	0	0	10.00	no unit80.00	no unit43.11	no unit2.10	23.49	no unit2249	counts	1166580.25	25.940	58329.02

[Cut]
[Slit Correction]
[LP Corr.]

[Absorp. Corr.]
[Norm.]
[Smoothing] Method : Averaging Smoothing Points: 11
[BG subtraction] Method : Averaging at the end
Low angle : 5 High angle : 12
[Ka2 elimination]

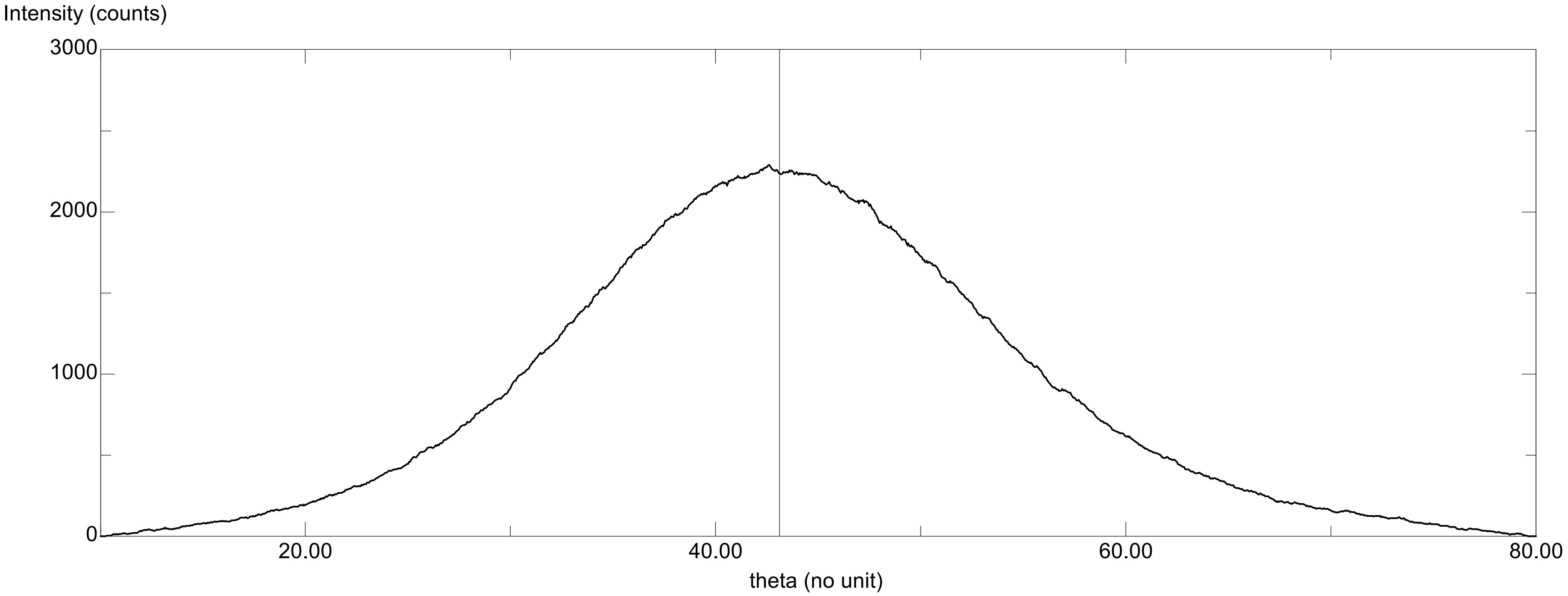

Integral int. sum table

Sample Name: File : 222.raw Date : May-17-24 10:23:12 Operator : Administrator
Memo :
Peak calc. method: FW2/3M

No.	h	k	l	Start	Stop	Peak	d-value	FWHM	Max. int.	Gross int.	Integ.w	Integ. int.	Comment
1	0	0	0	10.00	80.00	43.11	2.10	23.49	2249	1166580.25	25.940	58329.02	
